# Schwannoma of the nasal septum: evaluation of unilateral nasal mass

**DOI:** 10.5935/1808-8694.20130070

**Published:** 2015-10-04

**Authors:** Henrique Furlan Pauna, Guilherme Machado de Carvalho, Alexandre Caixeta Guimarães, Rebecca Christina Kathleen Maunsell, Eulália Sakano

**Affiliations:** a MD (ENT Resident Physician at UNICAMP).; b MSc in Medicine (MD, ENT (otology fellow at UNICAMP)).; c MSc in Medical Sciences (MD, ENT at the Otorhinolaryngology Service in the Sumaré State Hospital, ENT-HNS Course, UNICAMP).; d PhD in Medical Sciences (MD, ENT, Head of the Rhinology Service, ENT-HNS Course, UNICAMP). Otorhinolaryngology and Head and Neck Program - University Hospital - Medical School of the State University of Campinas (UNICAMP), SP, Brazil.

**Keywords:** nasal septum, neuroma, acoustic, otorhinolaryngologic neoplasms

## INTRODUCTION

Schwannomas are benign tumors originated in the neural crests derived from Schwann cells. They are often encapsulated, well circumscribed, and connected to nerve tissue. In microscope examination they present a combination of two growth patterns, Antoni A and B, both with elongated cells and regular oval nuclei. Malignant schwannomas occur in only 2% of the cases, although local recurrence is common in cases of incomplete tumor resection^1^.

The vestibulocochlear nerve ranks first among involved sites, accounting for 80% of the cases. Only 4% of the tumors of the head and neck are found in the nose or sinuses^2^. Autonomic nerve fibers or the maxillary branch of the trigeminal nerve may originate in the nose^2^. Schwannomas are more commonly seen in the ethmoid sinus, followed by the maxillary sinus, nasal cavities, and sphenoidal sinus^1^. Unilateral or bilateral progressive nasal obstruction, accompanied or not by epistaxis, hyposmia, and headaches, are usually observed in schwannoma patients. Physical examination usually reveals a grayish vegetative polypoid highly vascularized bleeding tumor. Examination with a fiberscope and CT scans are required to assess the extent of tumor involvement and plan for surgery^2^.

CT scans of paranasal schwannomas usually show low-density areas in their center with contrast uptake in the borders^3^.

This paper described the evolution of a patient with a unilateral tumor diagnosed as a schwannoma of the nasal septum.

## CASE REPORT

A 78-year-old male patient was referred to our service with progressive nasal obstruction evolving for two years.

Physical examination was normal and endoscopic examination of the nose revealed an exophytic grayish tumor with uneven borders, with a pedicle in the posterior septum region close to the upper corner of the right choana.

**Figure 1:**
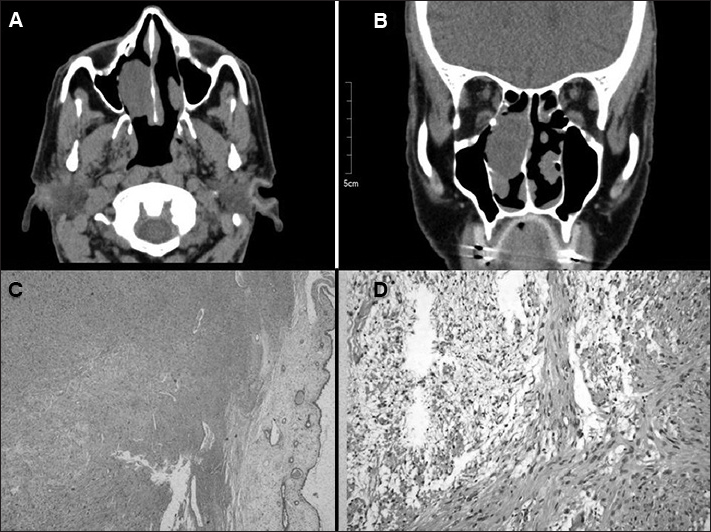
A: CT scan (axial view) showing a tumor in the right nasal cavity inserted in the posterior nasal septum. B: CT scan (coronal view) showing the tumor inserted in the septum projecting into the lower turbinate. C: Histology slide showing a myxoid formation to the right of the respiratory epithelium in the central portion of the image (lower cell count) characteristically seen in Antoni B tissue; spindle-shaped cells seen in Antoni A tissue are shown on the upper left corner. (HE;26.5x). D: Antoni B tissue s shown on the left (scarce cytoplasm with intercellular hydrated material) and Antoni A on the right of the slide (elongated cells crossing each other in bundles, with nuclei resembling a palisade formation). (HE;64x).

CT scans showed a nodular, low-density, well-delimited homogeneous tumor and some bone thinning in the medial wall of the right maxillary sinus (Figure 1).

The patient was submitted to endoscopic endonasal surgery. The tumor was removed with margins from the posterior septum periosteum. The procedure was uneventful.

Pathology tests showed proliferation of spindle-shaped cells arranged in a short fascicle pattern with oval wavy nuclei and acidophilic indistinct cytoplasm, characteristically seen in schwannomas (Figure 1).

## DISCUSSION

The literature comprises approximately 70 case reports of nasal schwannoma. Most cases involve adults in their forties to sixties, with little difference between genders. Complaints usually revolve around nasal obstruction, anosmia, deformity of the nasal pyramid, and epistaxis^2^.

CT scans usually show lesions in the surrounding bone structures; erosion is more commonly seen in large schwannomas. MRI outperforms CT in differentiating tumors from inflammatory disorders and normal tissue, in addition to providing better information on tumor intracranial invasion^3^.

Differential diagnosis for unilateral tumors includes nasal polyps (22.2%), antrochoanal polyps (19%), chronic rhinosinusitis (12.7%), concha bullosa (11.1%), retention cysts (6.3%), mucocele (3.2%), and schwannomas in 1.6% of the cases^4^. Other diseases considered are carcinoma, sarcoma, lymphoma, juvenile angiofibroma, inverted papilloma, meningioma, neurofibroma, melanoma, and olfactory neuroblastoma (esthesioneuroblastoma). Immunohistochemistry aids in the diagnosis of controversial cases.

Unilateral tumors must be thoroughly investigated, ideally with biopsy, particularly to diagnose cases of malignancy^4^. Preoperative biopsy was not carried out in our patient because of his age and the benign aspect of his tumor.

Treatment is mostly curative and can be offered in the form of surgical removal by lateral rhinotomy or endoscopic endonasal surgery^3^. Radiation therapy is reserved for cases of nerve sheath malignant tumors. Patients with tumors confined to the paranasal sinuses have excellent prognosis^5^. Endoscopic surgery introduces less morbidity, patients are not left with visible surgical scars, and the hospital stay after surgery is shorter^6^.

## CLOSING REMARKS

The diagnosis and management of patients with schwannoma can be challenging, as clinical and imaging findings of unilateral tumors may be deceiving. Schwannomas must be regarded as one of the possible diagnoses in such cases. Endoscopic endonasal surgery provides for a less invasive approach and offers an improved procedure to completely remove the tumor.
